# Creatinine, total cysteine and uric acid are associated with serum retinol in patients with cardiovascular disease

**DOI:** 10.1007/s00394-019-02086-2

**Published:** 2019-09-09

**Authors:** Thomas Olsen, Kathrine J. Vinknes, Rune Blomhoff, Vegard Lysne, Øivind Midttun, Indu Dhar, Per M. Ueland, Gard F. T. Svingen, Eva K. R. Pedersen, Christian A. Drevon, Helga Refsum, Ottar K. Nygård

**Affiliations:** 1 Department of Nutrition, Institute of Basic Medical Sciences, Faculty of Medicine, University of Oslo, Domus Medica, Sognsvannsveien 9, 0372 Oslo, Norway; 2 Department of Clinical Service, Division of Cancer Medicine, Oslo University Hospital, Forskningsveien 2A, 0372 Oslo, Norway; 3 Centre for Nutrition, Department of Clinical Science, University of Bergen, Haukelandsbakken, 5009 Bergen, Norway; 4 Bevital AS, Jonas Lies vei 87, 5021 Bergen, Norway; 5 Department of Heart Disease, Haukeland University Hospital, Jonas Lies vei 65, 5021 Bergen, Norway; 6 Mohn Nutrition Research Laboratory, Department of Clinical Science, University of Bergen, Haukelandsbakken, 5009 Bergen, Norway

**Keywords:** Retinol, Vitamin A, Cardiovascular disease, Creatinine, Cysteine, Uric acid

## Abstract

**Purpose:**

We hypothesized that biomarkers and dietary factors related to cardiovascular disease risk were associated with serum retinol and evaluated these potential associations in patients with suspected coronary artery disease (CAD).

**Methods:**

We used cross-sectional data from 4116 patients hospitalised for suspected CAD. Dietary data were obtained from a subgroup of 1962 patients using a food frequency questionnaire. Potential biomarkers and dietary factors were explored using linear regression modelling adjusted for age and sex. Regression coefficients and corresponding confidence intervals (CI) are given as  % change in serum retinol per unit change in the predictors. Analyses were performed in the total population and in strata of serum retinol tertiles.

**Results:**

In age- and sex-adjusted models, serum creatinine (standardized β: 0.38, 95% CI [0.35, 0.42]), plasma total cysteine (0.26, [0.23, 0.29]), serum uric acid (0.30, [0.26, 0.33]) and plasma neopterin (0.22, [0.18, 0.25]) were positively associated, whereas plasma serine (− 0.15, [− 0.18, − 0.12]) and serum C-reactive protein (− 0.15, [− 0.18, − 0.12]) were inversely associated with serum retinol. When we included the significant biomarkers in a multivariate model, the model explained 33% of the variability (*R*^2^ = 0.33) in serum retinol. The results were similar in the lower and upper tertiles of serum retinol. Weak or no associations were observed for dietary factors.

**Conclusions:**

In patients with suspected CAD, concentrations of creatinine, cysteine and uric acid were positively associated with serum retinol. Future studies should assess whether retinol concentrations are influenced by metabolic alterations in patients at risk of cardiovascular disease.

## Introduction

Vitamin A is an essential, fat-soluble micronutrient that refers to all-*trans* retinol and its bioactive metabolites retinaldehyde, retinoic acid (RA) as well as retinyl ester and pro-vitamin A carotenoids [1]. Following dietary intake from foods of plant and/or animal origin, retinol is transported as retinyl esters with triacylglycerol-rich chylomicrons to the liver where it can either be stored in hepatic stellate cells or exported to peripheral tissue bound to retinol-binding protein 4 (RBP4) and transthyretin. Retinol is then converted to RA in target tissues where it functions as a ligand for nuclear RA receptors with several target genes involved in growth and differentiation, metabolism of macronutrients [2], and the immune system [3].

We have recently reported that elevated serum retinol potentiates the risk of incident acute myocardial infarctions associated with traditional risk markers in subjects hospitalised for suspected coronary artery disease, including lipids and total homocysteine [4, 5]. The circulating retinol concentrations in these patients exceed those reported from other cohorts [6–9], and retinol concentrations above clinical reference ranges have been associated with the metabolic syndrome [10] and cardiovascular disease (CVD) [11]. In addition, plasma concentrations of RBP4, which circulates with retinol in a nearly 1:1 manner, were elevated in conditions characterized by metabolic dysfunction such as diabetes type 2 [12], obesity [13] and atherosclerosis [14].

In spite of these recent observations, biomarkers associated with circulating retinol concentrations have not been well characterized in populations with CVD. Although early findings suggest that serum retinol is under tight homeostatic control except during conditions of deficiency or extreme excess [15], recent reports suggest that patients with chronic kidney disease have higher [16], whereas inflammation may reduce [17] serum retinol. Thus, the aim of this exploratory cross-sectional study was to identify biomarkers and dietary factors associated with serum retinol in a large cohort of patients with suspected or established CVD. Our focus was primarily on biomarkers relevant for CVD risk including circulating lipid parameters, homocysteine and inflammatory markers, as well as dietary factors and kidney function. Analyses were conducted for the total population and following stratification of patients according to tertiles of serum retinol.

## Methods

### Study design

The study population has been described extensively elsewhere [18]. Briefly, 4164 patients were initially included in this study. All patients were recruited upon planned angiography for suspected stable angina pectoris at Haukeland (*n* = 3413) and Stavanger (*n *= 751) University Hospitals, Norway and 61.8% (*n *=2573) were enrolled in the Western Norway B-vitamin Intervention Trial (WENBIT) (clinicaltrials.gov: NCT00354081) [19]. The main aim of the WENBIT was to address the effect of homocysteine-lowering therapies with B-vitamins on a composite outcome consisting of all-cause death, nonfatal acute myocardial infarction, acute hospitalization for unstable stable angina pectoris and nonfatal thromboembolic stroke. Data were collected between 2000 and 2004. Patients with missing data on serum retinol (*n* = 46) and with extremely low (< 0.8 μmol/L) (*n *=1) or high (> 9.0 μmol/L) (*n* = 1) concentrations of retinol were excluded from the study, yielding a total of 4116 eligible patients for analysis. Informed consent, ethical approval, and necessary permissions from the Norwegian Medicines Agency and the Norwegian Data Inspectorate were obtained. The study was carried out according to the Declaration of Helsinki.

### Baseline data, biochemical analyses and food frequency questionnaire

Acquisition of clinical and diagnostic data including information on the presence and extent of coronary artery stenosis as determined by coronary angiography, body composition and smoking habits have been described in detail previously [18]. In addition, mostly non-fasting blood samples (80%) were collected at baseline and serum samples were stored at − 80 °C until analysis. Plasma concentrations of methionine, total homocysteine, cystathionine and cysteine were analysed using gas chromatography-mass spectrometry [20, 21], whereas serum all-*trans* retinol [22], creatinine [23] and plasma neopterin [24] were measured by high-performance liquid chromatography/tandem mass spectrometry. Kynurenine, tryptophan, 4-pyridoxic acid (PA), pyridoxal (PL) and pyridoxal-5-phosphate (PLP) were measured by liquid chromatography/tandem mass spectrometry [24]. Kynurenine to tryptophan ratio (KTR) and the PA-ratio (PAr; PA/PL + PLP) were calculated, and both have been proposed as markers of inflammation [25, 26]. All of the above measurements were performed in collaboration with Bevital AS, a company specializing in the assessment of biomarkers related to nutritional status and inflammation (http://www.bevital.no). Concentrations of fibrinogen, total cholesterol, apolipoprotein A1 and B, C-reactive protein (CRP), and dietary vitamin A intake were measured as described previously [4, 18, 27]. Serum uric acid measurements were part of the routine laboratory analytical panel [18].

Baseline dietary data were obtained from 2068 WENBIT participants from a 169-items food frequency questionnaire (FFQ). After exclusion due to noncompletion of the FFQ, very high (> 15,000 kJ/day for females, > 17,500 kJ/day for males) or very low energy intake (< 3000 kJ/day for females, 3500 kJ/day for males), 1962 patients were eligible for analysis. The development of the FFQ has been described elsewhere and has been validated for total energy intake and several nutrients [28–30]. Frequency of consumption and measures were converted by a conversion system developed by the Department of Nutrition, University of Oslo (Kostberegningssystem, version 3.2; University of Oslo). Vitamin A intake is given as retinol activity equivalents (RAE), and conversion values are based on the official Norwegian Food Table.

### Statistical analysis

The majority of the continuous data were not normally distributed. Log-transformed continuous variables are, therefore, presented as geometric means (gM) (geometric standard deviations [gSD]). Intake data on macronutrients are presented as the proportion of total energy intake (E %), and food items as densities (g/1000 kcal). Vitamin A intake is given as RAE/1000 kcal. Categorical variables are given as *n* (%). Baseline characteristics are given for the total population.

We used ordinary least squares regression to evaluate individual predictors of log-transformed concentrations of serum retinol. All models were adjusted for age (continuous) and sex (categorical) unless otherwise specified, and plasma/serum predictors were log-transformed. Models including dietary intakes were additionally adjusted for energy intake. No data transformation was applied to the dietary data. Estimates are reported as standardized β which indicate the standard deviation change in serum retinol per standard deviation change in the exposure variable, which is equivalent to the partial correlation coefficient.

For the sake of interpretability, we also report unstandardized *β*s (95% confidence interval). In models where the exposure and outcomes are log-transformed, the *β*s represent the  % change in serum retinol indicated by 1% increase in the exposure variable, respectively. These were subsequently multiplied by ten, and thus represent the  % change in serum retinol indicated by 10% increase in the exposure variable. For models including dietary data, the *β* represents the  % change in serum retinol indicated by 1 E % (macronutrients), 50 g/1000 kcal (food groups) or 200 RAE/1000 kcal. Additionally, we calculated the adjusted *R*^2^ to obtain the variance explained by each model. The linearity of associations was visualized using generalized additive model (GAM) plots. After Bonferroni adjustment, *p* < 0.001 was considered statistically significant. Finally, all variables associated with serum retinol after Bonferroni adjustment were included in a single model and the adjusted *R*^2^ was calculated. Statistical analyses were performed using R version 3.6.1 and packages included in the “tidyverse” as well as “sjstats”. All figures were made with the “ggplot2” package.

## Results

### Baseline characteristics

Baseline characteristics and dietary intake for the total population are presented in Table 1 and 2. Serum concentrations of retinol ranged from 1.02 to 7.65 μmol/L, and the gM (gSD) was 2.84 (1.26) μmol/L. The cohort consisted of 71.9% men, and gM (gSD) age was 60.8 (1.19) years, BMI was 26.5 (1.16) kg/m^2^ and creatinine was 90.2 (1.22) μmol/L. A total of 25.9% were smokers, whereas 74.8% had significant coronary stenosis at baseline angiography. For the 1962 patients that completed the FFQ, mean (SD) energy intake was 2073 (694) kcal whereas vitamin A intake was 945 (606) RAE/1000 kcal/day.

**Table 1:** Baseline characteristics of the total population (*n* = 4116)

Retinol, μmol/L	2.80 (1.26)
Age, years	60.8 (1.20)
Male sex, *n* (%)	2997 (71.9)
Smokers, *n* (%)	1321 (31.7)
Body mass index, kg/m^2^	26.5 (1.16)
Lipid parameters	
Apolipoprotein B, g/L	0.87 (1.31)
Apolipoprotein A1, g/L	1.29 (1.23)
Triglycerides, mmol/L	1.54 (1.67)
Homocysteine metabolism, μmol/L	
Methionine	27.0 (1.30)
Total homocysteine	10.7 (1.38)
Cystathionine	0.28 (1.81)
Total cysteine	290 (1.14)
Serine	95.9 (1.26)
Glycine	211 (1.29)
Inflammation	
C-reactive protein, mg/mL	3.64 (2.43)
Uric acid, μmol/L	347 (1.28)
Neopterin, nmol/L	8.57 (1.47)
Fibrinogen, g/L	3.61 (1.21)
KTR	2.43 (1.37)
PAr	0.51 (1.58)
Extent of CVD	
1–3 stenotic vessels, *n* (%)	3120 (74.9)
Previous acute myocardial infarction, *n* (%)	1680 (40.3)
Ejection fraction < 60%, *n* (%)	3277 (78.7)
Kidney function	
Creatinine, μmol/L	90.2 (1.22)

Baseline characteristics of the total population. Continuous variables are presented as geometric means and geometric standard deviations. Categorical variables are presented as count and per cent

**Table 2:** Baseline nutrient and food intake

Nutrients, E %	Mean (SD)
Vitamin A, RAE/1000 kcal	945 (606)
Protein	16.7 (2.55)
Carbohydrate	48.9 (6.46)
Total fat	31.9 (5.61)
PUFA	7.21 (1.98)
MUFA	10.3 (2.03)
SFA	11.8 (2.65)
Alcohol	2.06 (3.16)
Foods, g/1000 kcal	
Meat	54.8 (23.4)
Vegetables	105 (80.9)
Fruits and berries	124 (86.1)
Eggs	8.38 (6.30)
Dairy	154 (112)
Fish	53.7 (28.8)

Baseline mean and standard deviation intake of nutrients and food groups

### Biomarkers associated with retinol in the total population

**Fig. 1:**
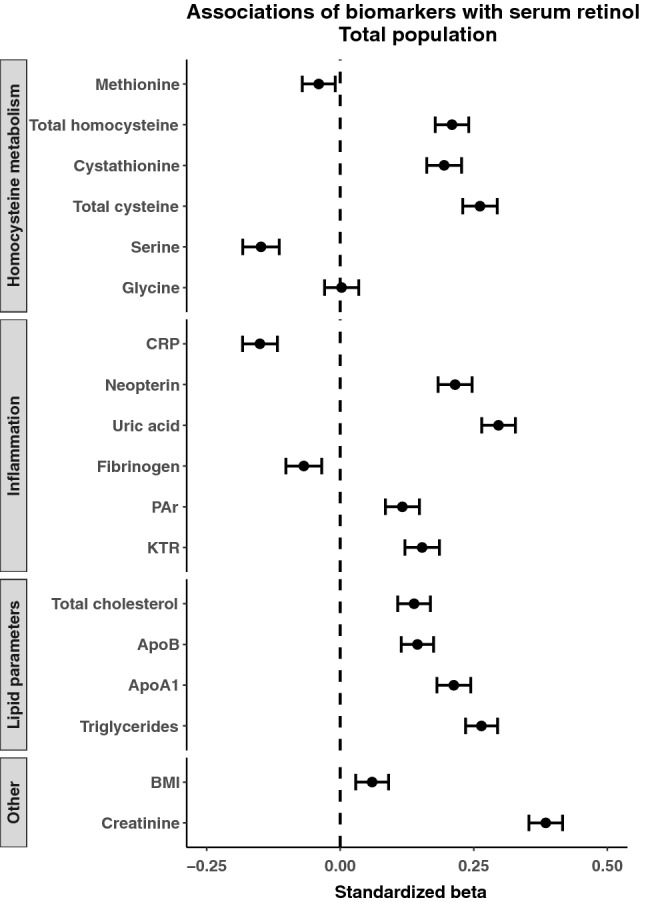
Forest plot of predictors of serum retinol. The standardized β and corresponding confidence intervals were derived from linear regression models adjusted for age and sex. *CRP* C-reactive protein, *apo* apolipoprotein, *KTR* kynurenine to tryptophan ratio, *PAr* pyridoxic acid to pyridoxal + pyridoxal-5-phosphate ratio, *BMI* body mass index

All analyses are presented in Fig. 1. Regression coefficients and their confidence intervals are given in Table 3. The strongest associations were found for serum creatinine, uric acid, triglycerides and plasma total cysteine, all of which were positively associated with serum retinol. Other positively associated biomarkers included plasma neopterin, plasma total homocysteine and serum apolipoprotein A1. Negatively associated biomarkers included CRP and plasma serine. Because serum creatinine is used as a marker of kidney function, which may confound associations between biomarkers and serum retinol in the circulation, additional models were created and adjusted for serum creatinine. The results remained particularly for plasma total cysteine (standardized *β* = 0.17, 95% CI: 0.13, 0.20, *p* < 0.001), serum uric acid (standardized *β* = 0.20, 95% CI: 0.17, 0.24, *p* < 0.001) and plasma serine (standardized *β* = − 0.11, 95% CI: − 0.14, − 0.07) and serum retinol, whereas the remaining associations were severely attenuated (data not shown).

**Table 3:** Regression coefficients and confidence intervals for biomarkers associated with retinol

	Standardized *β* (95% CI)	*β* per 10% increase (95% CI)	*p* value	Adjusted *R*^2^ (%)
Lipid parameters				
Total cholesterol	0.14 (0.11, 0.17)	1.47 (1.15, 1.8)	< 0.001	2.17
Apolipoprotein B	0.14 (0.11, 0.17)	1.37 (1.04, 1.51)	< 0.001	2.36
Apolipoprotein A1	0.21 (0.18, 0.24)	2.44 (2.12, 2.81)	< 0.001	4.36
Triglycerides	0.26 (0.23, 0.29)	1.22 (1.10, 1.32)	< 0.001	7.08
Homocysteine metabolism				
Methionine	− 0.04 (− 0.07, − 0.01)	− 0.36 (− 0.62, − 0.11)	0.007	0.44
Total homocysteine	0.21 (0.18, 0.24)	1.52 (1.29, 1.75)	< 0.001	4.29
Cystathionine	0.19 (0.16, 0.23)	0.76 (0.21, 0.52)	< 0.001	3.80
Total cysteine	0.26 (0.23, 0.29)	4.81 (4.21, 5.32)	< 0.001	7.11
Serine	− 0.15 (− 0.18, − 0.11)	− 1.52 (− 1.88, − 1.17)	< 0.001	2.17
Glycine	0.00 (− 0.03, 0.30)	0.03 (− 0.27, 0.31)	0.823	0.30
Inflammation				
C-reactive protein	− 0.15 (− 0.18, − 0.12)	− 0.41 (− 0.49, − 0.32)	< 0.001	2.50
Uric acid	0.30 (0.26, 0.33)	2.81 (2.52, 3.23)	< 0.001	9.71
Neopterin	0.22 (0.18, 0.25)	1.43 (1.21, 1.62)	< 0.001	4.34
PAr	0.12 (0.08, 0.15)	0.60 (0.43, 0.76)	< 0.001	1.53
KTR	0.15 (0.12, 0.19)	1.14 (0.90, 1.38)	< 0.001	2.35
Fibrinogen	− 0.07 (− 0.10, − 0.04)	− 0.83 (− 1.25, − 0.42)	< 0.001	0.70
Body mass				
Body mass index	0.06 (0.029, 0.09)	0.97 (0.47, 1.47)	0.013	0.60
Kidney function				
Creatinine	0.38 (0.35, 0.42)	4.51 (4.19, 4.93)	< 0.001	14.5

Regression coefficients for various predictors. All models were adjusted for age and sex. Coefficients represent the percentage change in retinol per 10% increase in the predictor and the adjusted *R*^2^ represents the predictive power of the models

*PAr* 4-pyridoxic acid/pyridoxal + pyridoxal-5-phophate ratio, *KTR* kynurenine/tryptophan ratio

**Fig. 2:**
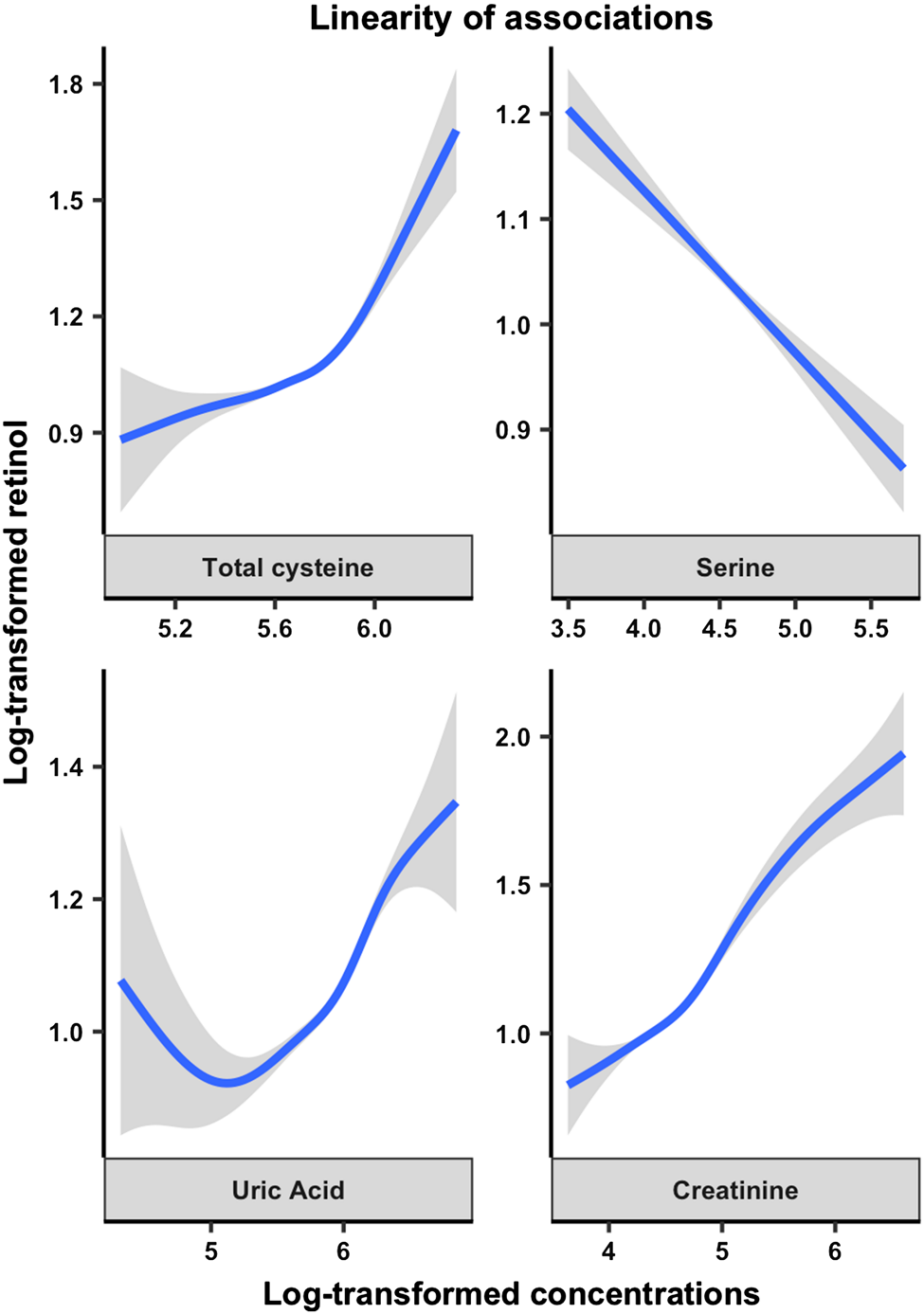
Generalized additive model plots of the age and sex-adjusted linear association between selected predictors and log-transformed serum retinol

We evaluated the linearity of the associations by fitting GAM-curves for the associations of serum creatinine, uric acid, plasma total cysteine and serine with serum retinol as shown in Fig. 2. All associations appeared to be linear.

**Fig. 3:**
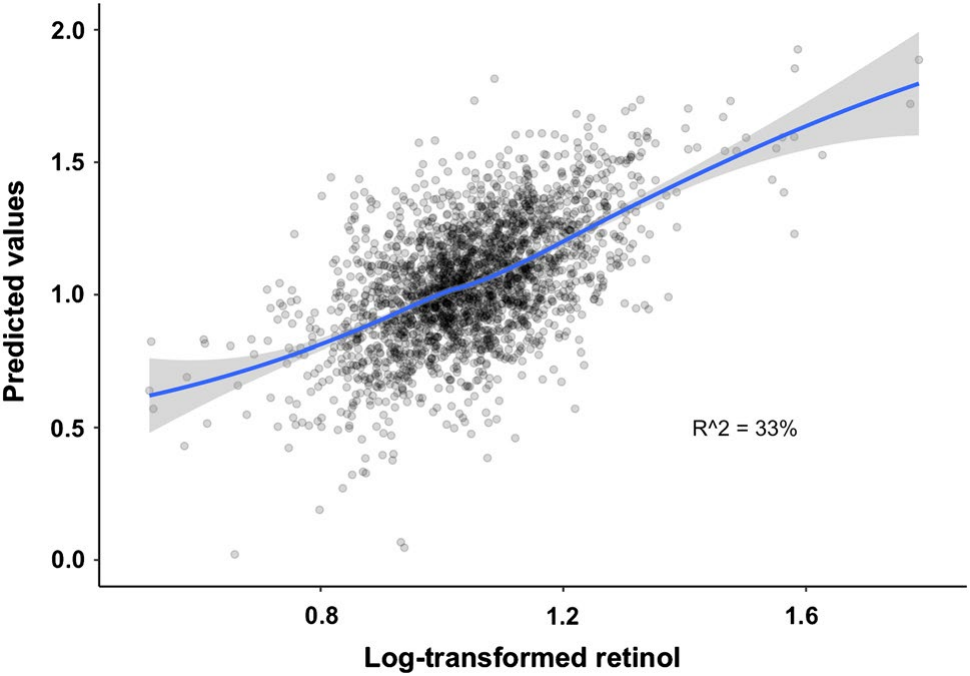
Observed vs. predicted values of log-transformed serum retinol derived from a linear regression model including age, sex, total cysteine, uric acid, creatinine, neopterin, total cholesterol, apolipoprotein A1 and B, triglycerides, total homocysteine and cystathionine, fibrinogen, pyridoxic acid to pyridoxal + pyridoxal-5-phosphate ratio and kynurenine to tryptophan ratio

To assess the fraction of variance explained by all associated biomarkers after Bonferroni adjustment, we included age, sex, serum creatinine, total cholesterol, apolipoprotein A1 and B, triglycerides, uric acid and CRP, and plasma total homocysteine, cystathionine, total cysteine, serine, KTR, PAr, fibrinogen and neopterin in a multivariate regression model and calculated the adjusted *R*^2^. The total proportion of variance explained was 33%, and the predicted vs. observed values of log-transformed retinol based on this model are illustrated in Fig. 3.

### Associations between dietary intakes and serum retinol

In total, 1962 patients completed the FFQ, and associations of dietary factors and retinol in serum are presented in Table 4. A weak, positive association was observed for meat and vegetable intake with serum retinol, whereas no particular associations were observed for other dietary variables including energy-adjusted RAE intake.

**Table 4:** Regression coefficients and confidence intervals for dietary predictors of serum retinol

Macronutrients	Standardized *β* (95% CI)	*β* per *E* % (95% CI)	*p* value	Adjusted *R*^2^ (%)
Protein	0.063 (0.02, 0.11)	0.52 (0.16, 0.89)	0.005	1.10
Carbohydrate	− 0.062 (− 0.11, − 0.018)	− 0.21 (− 0.36, − 0.07)	0.004	1.18
Total fat	0.002 (− 0.041, 0.045)	− 0.02 (− 0.19, 0.14)	0.77	0.70
PUFA	− 8e− 04 (− 0.045, 0.043)	− 0.11 (− 0.58, 0.36)	0.65	0.79
MUFA	0.022 (− 0.022, 0.065)	0.15 (− 0.31, 0.61)	0.53	0.83
SFA	− 0.0053 (− 0.048, 0.038)	− 0.12 (− 0.47, 0.22)	0.48	0.81
Alcohol	0.077 (0.026, 0.13)	0.47 (0.15, 0.79)	0.004	1.01
Micronutrient		**β per 200 RAE/1000 kcal (95% CI)**		
Vitamin A	0.017 (− 0.029, 0.062)	0.29 (− 0.03, 3.27)	0.09	0.80
Foods		**β per 50 g/1000 kcal (95% CI)**		
Meat	0.076 (0.032, 0.12)	3.60 (1.54, 5.66)	< 0.001	1.30
Vegetables	0.04 (− 0.0033, 0.084)	0.91 (0.33, 1.50)	0.002	1.15
Fruits and berries	0.005 (− 0.039, 0.049)	0.13 (− 0.41, 0.68)	0.63	0.70
Eggs	0.012 (− 0.032, 0.056)	0.55 (− 7.13, 8.25)	0.89	0.70
Dairy	0.048 (0.0048, 0.092)	0.41 (− 0.01, 0.82)	0.06	0.91
Fish	− 0.0068 (− 0.051, 0.037)	− 0.02 (− 1.67, 1.61)	0.97	0.72

Regression coefficients for various dietary predictors. All models were adjusted for age, sex and energy intake. Coefficients represent the percentage change in vitamin A per 10% increase in the predictor and the adjusted *R*^2^ represents the predictive power of the models

### Factors associated with retinol in patients with low and high concentrations

**Fig. 4:**
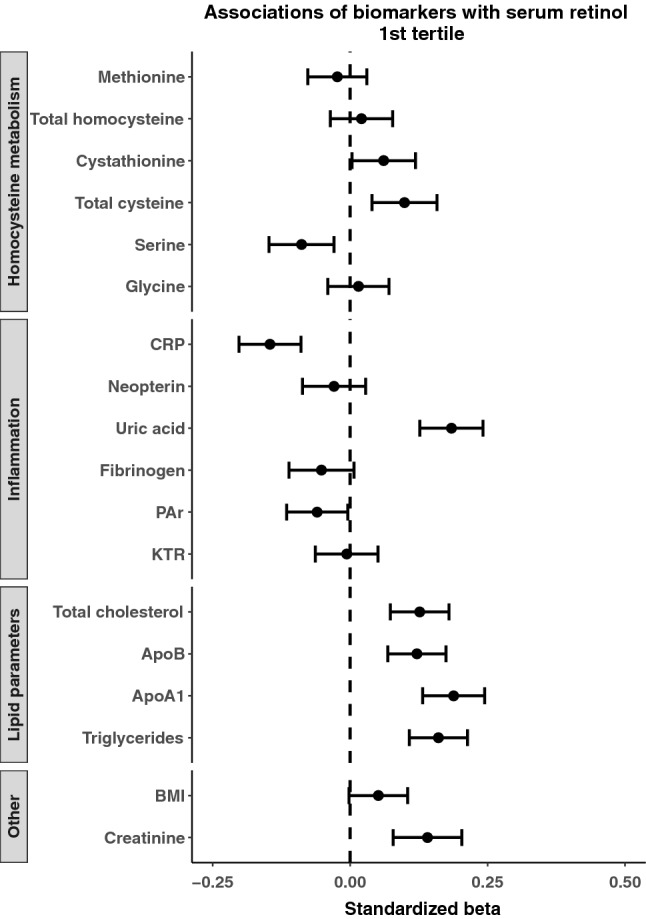
Forest plot illustrating predictors of serum retinol in the lower retinol tertile. The standardized *β* and corresponding confidence intervals were derived from linear regression models adjusted for age and sex. *CRP* C-reactive protein, *apo* apolipoprotein, *KTR* kynurenine to tryptophan ratio, *PAr* pyridoxic acid to pyridoxal + pyridoxal-5-phosphate ratio, *BMI* body mass index

**Fig. 5:**
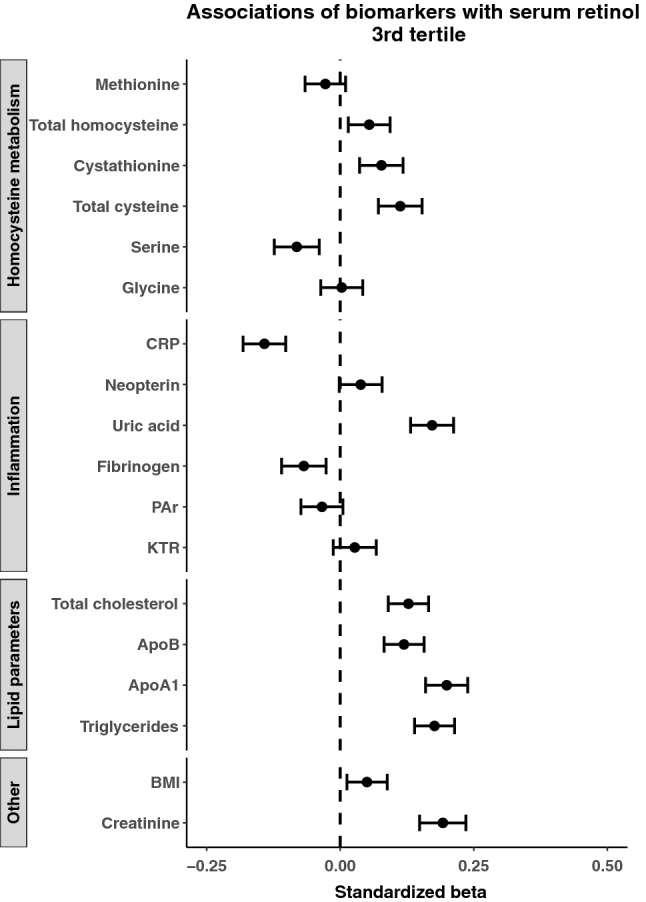
Forest plot illustrating predictors of serum retinol in the upper retinol tertile. The standardized *β* and corresponding confidence intervals were derived from linear regression models adjusted for age and sex. *CRP* C-reactive protein, *apo* apolipoprotein, *KTR* kynurenine to tryptophan ratio, *PAr* pyridoxic acid to pyridoxal + pyridoxal-5-phosphate ratio, *BMI* body mass index

Because of the substantial range in serum concentrations of retinol (1.02–7.65 μmol/L) we explored potential predictors, separately in patients in the lower and higher ranges of serum retinol. Results according to serum retinol tertiles are presented in Figs. 4 and 5. Results from the 1st and 3rd tertile (retinol < 2.57 and > 3.08 μmol/L, respectively) showed generally the same, but slightly weaker associations as for the total population. Associations for serum creatinine, plasma total cysteine and serum uric acid remained prominent in the 1st and 3rd tertiles. The associations for dietary intakes with serum retinol were essentially similar for the total population (data not shown).

## Discussion

### Principal findings

Serum retinol has generally been considered to be under tight homeostatic control [15], and we have previously shown that retinol in serum of patients hospitalised for suspected coronary artery disease may range beyond what has been observed in other cohorts [4–9]. Factors associated with this variation have not been elucidated to a meaningful extent in CVD patients. In this exploratory study, we report observed associations for circulating and dietary factors with serum retinol in a large cohort of patients with suspected coronary artery disease. The most prominent associations were observed for creatinine, triglycerides, uric acid, serine and the sulphur amino acid cysteine. When we adjusted for serum creatinine, several of the associations were attenuated, whereas those of total cysteine and uric acid with serum retinol remained. Other positively associated biomarkers included plasma neopterin, but this association was attenuated after adjustment for creatinine. With the exception of meat and vegetable consumption, we found no particular associations between dietary intakes and serum retinol.

### Serum creatinine and retinol

We observed a positive association between serum creatinine, a marker of kidney function, and serum retinol. This is in line with previous findings showing that retinol in serum increases in chronic kidney disease [16, 31] and that estimated glomerular filtration rate is an important determinant of retinol in an elderly population [32]. One case–control study found that serum retinol was positively associated with hypertension, and the authors speculate that this effect might have been mediated by kidney dysfunction [33]. However, because the RBP4-retinol-transthyretin complex is too large to be filtered, the kidneys are not considered to be an important route of retinol excretion [1]. The association between serum creatinine and retinol may thus be affected by other factors, and not increased renal retention. One study suggests that elevated retinol in kidney dysfunction is due to disrupted signalling pathways that increase retinol release from liver storage when kidney function is compromised [16], indicating that metabolic alterations may affect vitamin A homeostasis with unknown consequences in patients with CVD.

### Plasma cysteine, serine and serum retinol

To our knowledge, this is the first study demonstrating associations of plasma total cysteine and serine with serum retinol. In metabolism, serine is produced from glycolysis, serve as a precursor for glycine [34], and may condense with homocysteine to produce cystathionine and ultimately cysteine in transsulfuration [35, 36]. In observational studies, plasma serine and total cysteine were inversely and positively associated to components of the metabolic syndrome, respectively [37–40]. Because there is little literature available on the possible relationship between cysteine and serine with retinol, it is difficult to interpret the direction of the observed associations in the present study, but some evidence from other populations with lifestyle diseases suggest that retinol may affect the metabolism of these amino acids. Notably, higher circulating concentrations of retinol bound to RBP4 may be associated with dysregulated glucose metabolism [12] which can impact serine production from glycolysis and partly explain the observed inverse association between serine and retinol. Furthermore, animal models have demonstrated that enzymes involved in homocysteine and cysteine metabolism can be induced by RA administration [41–43]. Interestingly, both serine—as a glycine precursor—and cysteine are central to the hepatic formation of glutathione [35, 36, 44] a major antioxidant of which plasma concentrations can be low in subjects with obesity and CVD [45, 46]. Whether the possible effects of retinol on serine and cysteine influence glutathione status is not known, and future studies should thus seek to address whether elevated cysteine and reduced serine in lifestyle disease and CVD (1) reflect reduced glutathione synthesis and (2) whether this effect is mediated by the bioactive RA.

### Inflammatory markers and serum retinol

Studies on the relationship between inflammation and vitamin A are extensive [3, 17, 47]. Markers of the acute phase response, such as CRP, are inversely related to retinol [17]. It is generally accepted that systemic inflammation may contribute to increased sequestration of retinol in tissues and subsequently to reduced serum concentrations. In line with this notion, we observed an inverse association between CRP and retinol. In contrast, other inflammatory markers associated with CVD risk, such as uric acid [48], were positively associated with serum retinol. This particular finding reflects those of others, which have shown a positive association between uric acid and retinol in large and healthy cohorts [49, 50]. There may be several potential unmeasured factors that affect this association, but interestingly, the enzyme that produce uric acid—xanthine oxidase—has been linked to endothelial dysfunction in atherosclerosis [51] and can also catalyse the formation of RA [52]. Further, neopterin, a marker of the pro-atherogenic T_h_1 cell-mediated monocyte activation that has been linked CVD risk [53, 54] was positively associated with retinol in the present investigation. It is not known whether neopterin itself affects retinol concentrations, however, in vitro studies show that RA administered in physiological doses activate T_h_1 cells [47] which in turn can contribute to proliferation of monocytes into macrophages and increased neopterin concentrations [53]. Our findings indicate a potential interplay between inflammatory processes in atherosclerosis and retinol and should be addressed in future studies. Specifically, the uptake and metabolism of retinol and activity of RA in immune cells during inflammatory processes specific to atherosclerosis would provide insight in this context.

### Diet and serum retinol

The observed associations for the dietary predictors were weak, which is not unexpected considering the relatively stable concentrations of circulating retinol [55, 56]. Although the existing evidence for the association for meat intake and retinol is somewhat conflicting [57, 58] we did observe positive association between meat intake and serum retinol. Meat (in particular processed meat) intake should be limited in the context of CVD prevention [59] and taken together with the other results of the present investigation indicate that high serum concentrations of retinol at least in part are explained by factors related to an unfavourable risk profile in these patients. We cannot exclude the possibility that the bias present in dietary assessment tools obscured the true associations.

### Strengths and limitations

The major strength of our study was the large, well-characterized cohort including more than 4000 patients, the majority with angiographically verified coronary artery disease, which provided a solid basis for the evaluation of biomarkers and dietary factors associated with serum retinol. However, our findings cannot be generalized beyond the study population, because serum concentrations of retinol appear to be elevated in patients with CVD compared to a presumably healthy population residing in the same geographical area [60]. Further limitations include the cross-sectional design, which complicates the interpretation of the direction of the associations. Moreover, at this point the clinical implications are limited because many of the results presented here are novel, and relevance is difficult to establish until results are replicated in other cohorts. The interpretation of the associations between total cysteine and uric acid with retinol was particularly complicated because it is unclear whether cysteine and uric acid affect retinol or vice versa.

It should be noted that the proportion of variance explained by the models was generally low. Overall, the adjusted *R*^2^ varied from 4 to ~ 15%, for the models, whereas a multivariate model including the most strongly associated biomarkers explained about 33% of the total variation in serum retinol. Although some of the unmeasured variations may be attributed to genetics [61, 62], we emphasize that very little is currently known about factors affecting retinol in the circulation and that a continued effort should be undertaken to further explore this particular knowledge gap.

## Conclusion

Biomarkers associated with retinol in patients with established CVD include metabolites that are linked to metabolic disease, kidney function, and inflammation. Future observational and experimental studies should assess the potential causal direction and the clinical relevance of these associations. Finally, it would be useful to assess these associations in healthy populations, to further uncover the role of retinol in health and disease.
